# First record of the genus *Rasnitsynoryctes* Belokobylskij, 2011 (Hymenoptera, Braconidae, Doryctinae) in Vietnam, with the description of a new species

**DOI:** 10.3897/zookeys.854.34810

**Published:** 2019-06-10

**Authors:** Khuat Dang Long, Sergey A. Belokobylskij

**Affiliations:** 1 Institute of Ecology and Biological Resources (IEBR), Vietnam Academy of Science and Technology (VAST), 18 Hoang Quoc Viet Road, Ha Noi, Vietnam Institute of Ecology and Biological Resources, Vietnam Academy of Science and Technology Ha Noi Vietnam; 2 Zoological Institute of the Russian Academy of Sciences, Universitetskaya naberezhnaya 1, Saint Petersburg 199034, Russia Zoological Institute, Russian Academy of Sciences St. Petersburg Russia; 3 Museum and Institute of Zoology of the Polish Academy of Sciences, Wilcza 64, 00–679 Warsaw, Poland Museum and Institute of Zoology, Polish Academy of Sciences Warsaw Poland

**Keywords:** Ichneumonoidea, new record, Oriental region, parasitoid

## Abstract

The rare doryctine genus *Rasnitsynoryctes* Belokobylskij, 2011 is recorded for the braconid fauna of Vietnam for the first time. A new species of this genus, *R.vietnamicus***sp. nov.**, is described and illustrated.

## Introduction

The peculiar monotypic Oriental genus *Rasnitsynoryctes* Belokobylskij, 2011, with type species *Rasnitsynoryctesalexandri* Belokobylskij, 2011, is a rare taxon from subfamily Doryctinae originally described from Malaysia (Belokobylskij, 2011). The one of the most important features of this genus, presence of longitudinal and weakly convergent posteriorly sublateral furrows, is known in several others Old World genera: several taxa from the tribes Holcobraconini and Leptospathiini, *Eodendrus* Belokobylskij, 1998; *Hypodoryctes* Kokujev, 1900; *Halycaea* Cameron, 1903 and *Sonanus* Belokobylskij et Konishi, 2001 from Doryctini, *Polystenus* Foerster, 1863; *Spathiostenus* Belokobylskij, 1993 and *Terate* Nixon, 1943 from Hecabolini. *Rasnitsynoryctes* is additionally characterised by the fore wing with discal (discoidal) cell sessile anteriorly and vein CU1b (brachial) slanted towards base of wing (declivous); hind wing with subbasal (submedial) cell short and with more than three hamuli; inner spur of hind tibia transformed, sinuate and with inner expansion in apical third or submedially.

The hosts of the member from this genus are yet unknown. According to the large size of *Rasnitsynoryctes* specimens and by analogy to many other large-sized doryctines, species of this genus are probably parasitoids of Cerambycidae larvae or some other large xylophagous beetles inhabiting similar ecological niches.

In this paper we describe and illustrate the new species of the genus *Rasnitsynoryctes*, *R.vietnamicus* sp. nov., and additionally, this genus is recorded for the first time for the fauna of Vietnam.

## Materials and methods

The studied specimen is deposited in the Braconidae Collection of the Institute of Ecology & Biological Resources (IEBR), the Vietnam Academy of Science and Technology, Ha Noi, Vietnam.

Terminology used in this paper follows van Achterberg (1993), while sculpture terms are based on Harris (1979). The wing venation nomenclature follows van Achterberg (1993), with Belokobylskij and Maetô (2009) terminology shown in parentheses.

We used an Olympus SZ61 binocular microscope for study; measurement were carried out using an Olympus SZ40 binocular microscope; the photographs were made with a Sony 5000 digital camera attached to a Nikon SMZ 800N binocular microscope connected to a PC at IEBR and processed with Adobe Photoshop CS5 to adjust the size and background. Abbreviations used in this paper are as follows:

**POL** minimum postocellar line;

**OOL** minimum ocular-ocellar line;

**OD** maximum diameter of posterior ocellus;

**MT** Malaise trap;

‘**Doryc. + number**’ code number indexing for Doryctinae specimens in the collection at IEBR;

**NR** Nature Reserve.

## Taxonomy

### Genus Rasnitsynoryctes

Taxon classificationAnimaliaHymenopteraBraconidae

Belokobylskij, 2011

Rasnitsynoryctes Belokobylskij, 2011: 241.

#### Type-species.

*Rasnitsynoryctesalexandri* Belokobylskij, 2011.

#### Diagnostic characters.

Frons weakly concave. Eyes glabrous. Occipital carina dorsally complete, obliterate below at long distance above hypostomal carina. Malar suture absent. Postgenal bridge rather wide. Maxillary palpi long. Notauli complete. Precoxal sulcus narrow and long. Prepectal carina complete. Propodeum with finely delineated basolateral areas; lateral tubercles and propodeal bridge absent. Pterostigma of fore wing rather narrow. Marginal (radial) cell not shortened. Vein m-cu (recurrent) weakly antefurcal. Discal (discoidal) cell sessile anteriorly. Vein CU1a (parallel) arising from posterior 0.2–0.25 of apical margin of subdiscal (brachial) cell. Subdiscal (brachial) cell closed postero-apically by vein CU1b (brachial). Veins 2A and a (first and second transverse anal veins) absent. Hind wing with 5–6 hamuli. Marginal (radial) cell without additional transverse vein r. Subbasal (submedial) cell short; vein M+CU (first abscissa of mediocubital) 0.35–0.40 times as long as vein 1-M (second abscissa). Vein m-cu (recurrent) short, distinctly slanted toward base of wing. Fore tibia with short and thick spines arranged in almost single line. Hind coxa with distinct basoventral tooth. Hind tibia inner spur distinctly sinuate (Fig. 15) and with inner expansion in apical third. Basitarsus of hind tarsus 0.9–1.1 times as long as second-fifth segments combined. First metasomal tergite not petiolate, long and wide; acrosternite of first segment short, about 0.15 times as long as first tergite. Dorsope of first tergite large; spiracular tubercles situated in basal 0.25 of tergite. Second tergite with deep, weakly convergent posteriorly and fused with second suture sublateral furrows. Suture between second and third tergites rather deep, narrow, widely curved medially and laterally with distinct breaks. Second to sixth tergites with separate laterotergites. All tergites and laterotergites covered by very dense, short, white setae. Ovipositor apically with two obtuse, small dorsal nodes.

### Rasnitsynoryctes vietnamicus

Taxon classificationAnimaliaHymenopteraBraconidae

Long & Belokobylskij sp. nov.

http://zoobank.org/19483512-1317-41D9-BE8C-B2CC3F6BCB40

Figs 1 , 2–16

#### Type material.

Holotype, female, “Doryc. 673”, NE Vietnam: Bac Giang, Son Dong, Yen Tu NR, 300 m, 4.vii.2010 (PT Nhi leg.) (IEBR).

#### Comparative diagnosis.

The new species, *Rasnitsynoryctesvietnamicus* sp. nov., is very similar to the type species of the genus, *R.alexandri* Belokobylskij, 2011, from Malaysia; the differences between these species are showed in the key below after description.

**Figure 1.**
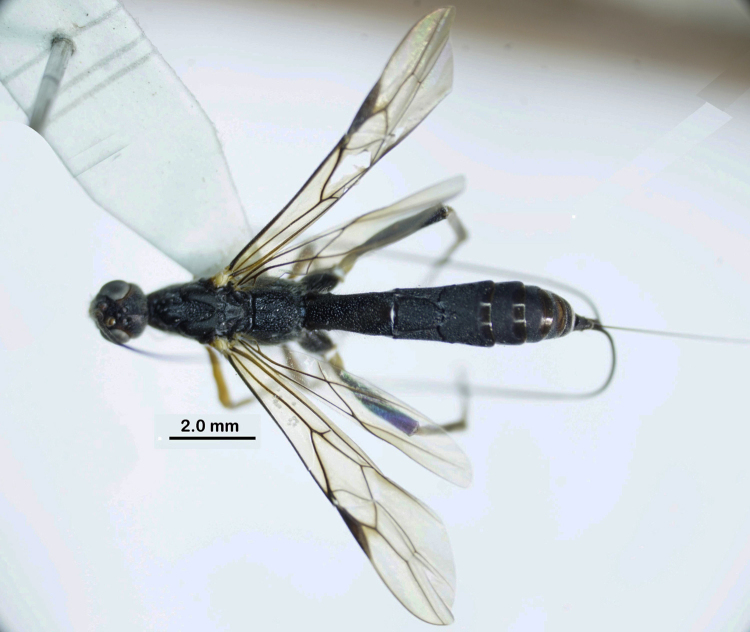
Habitus in dorsal view of *Rasnitsynoryctesvietnamicus* sp. nov., female, holotype.

#### Description.

Female. Body length 11.7 mm, fore wing length 8.6 mm, ovipositor sheath 12.0 mm (Fig. 1).

**Figure 2–16.**
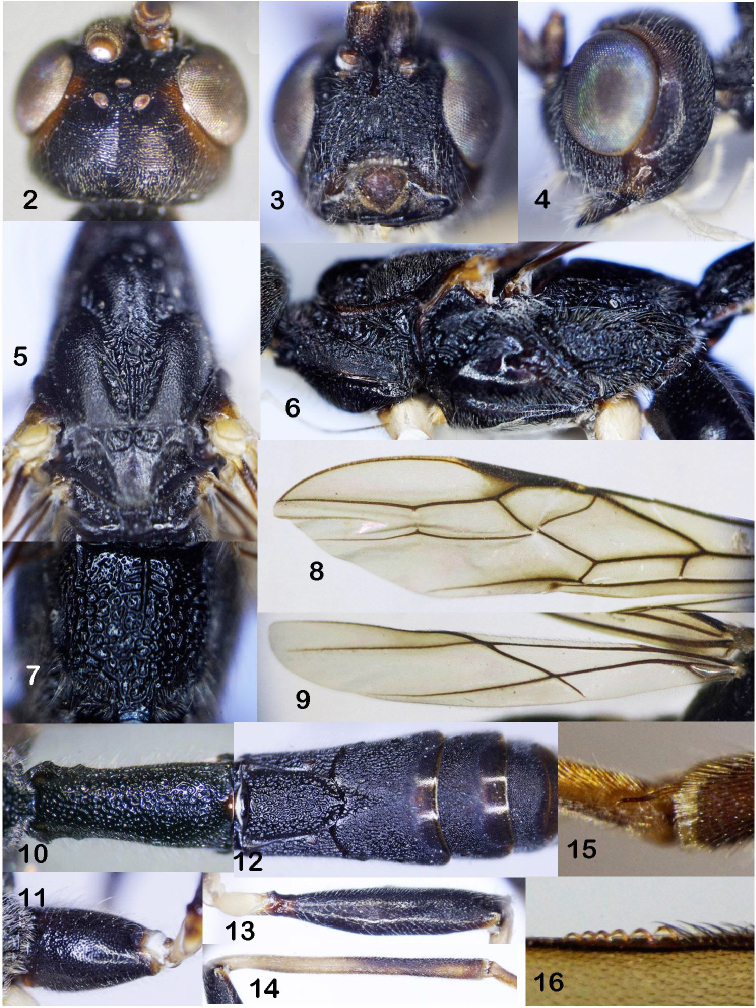
*Rasnitsynoryctesvietnamicus* sp. nov., female, holotype **2** head, dorsal view **3** head, front view **4** head, lateral view **5** mesonotum **6** mesosoma, lateral view **7** propodeum **8** fore wing **9** hind wing **10** first metasomal tergite **11** hind coxa, outer side **12** second-fifth metasomal tergites **13** hind femur, outer side **14** hind tibia, outer side **15** inner spur of hind tibia **16** hind wing hamuli.

*Head*. Antennae with more than 48 segments (apical segments missing); scapus 1.4 times as long as its maximum width (14 : 10); third segment almost as long as fourth segment (27 : 26); middle segments 3.5–3.7 times as long as their width. Head width (dorsal view) 1.2 times its median length (64 : 52), head roundly narrowed behind eyes (Fig. 2), length of eye 1.6 times as long as temple (44 : 28); ocelli rather small, POL : OD : OOL = 7 : 5 : 8; in lateral view, eye 1.4 times as long as temple (25 : 18) (Fig. 4); maxillary palp 1.8 times as long as height of head (103 : 57); face width 1.4 times length of face and clypeus combined (35 : 25) (Fig. 3); malar space 0.7 times basal width of mandible (11 : 15); width of hypoclypeal depression equal to distance from edge of depression to eye (15 : 15); distance between tentorial pits 1.45 times distance from pit to eye (16 : 11); occipital carina not fused below with hypostomal carina above base of mandible but almost faded with patch of coarse rugosities near base of mandible.

*Mesosoma*. Length 2.3 times its height (77 : 34); mesoscutum highly and perpendicularly elevated above pronotum; median lobe of mesoscutum with distinct median longitudinal depression (Fig. 5); notauli deep, crenulate anteriorly, widened posteriorly, coarsely rugose (Fig. 5); pronotal sides largely crenulate medially, granulate ventrally (Fig. 6); prescutellar depression with only median carina, 0.3 times as long as scutellum (6 : 19); subalar depression wide and deep, with oblique rugosity; precoxal sulcus long, shallow, almost smooth (Fig. 6); metapleuron setose, coarsely rugose; propodeum with median carina in basal 0.3 of propodeum, without delineated areola (Fig. 7).

*Wings*. Length of fore wing 4.25 times as long as its maximum width (102 : 24) (Fig. 8); vein 1-R1 (metacarp) 1.5 times as long as pterostigma (58 : 38); length of pterostigma 4.75 times its width (38 : 8); vein r (radial) omitting before middle of pterostigma, 0.6 times as long as vein 2-SR (first radiomedial) and 0.35 times as long as vein 3-SR (second radial abscissa); r : 2-SR : 3-SR : SR1 (third radial abscissa) = 9 : 16 : 26 : 42; basal length of second submarginal (second radiomedial) cell 2.8 times its maximum width (36 : 13), 1.1 times length of subdiscal (brachial) cell basally (36 : 32); vein 1-CU1 : cu-a (nervulus) : 2-CU1 = 5 : 6 : 28; vein m-cu (recurrent) oblique. Length of hind wing 5.5 times as long as its maximum width (77 : 14); vein M+CU (first mediocubital abscissa) short, 0.3 times as long as vein 1-M (second mediocubital abscissa) (Fig. 9); vein M+CU : 1-M : r-m (basal) = 16 : 58 : 30.

*Legs*. Inner side of fore tibia with row of short robust spines; middle basitarsus 15.0 times as long as its width (60 : 4) and 0.9 times as long as tarsal segments 2–4 combined (60 : 68); hind coxa 1.6 times as long as its maximum width (52 : 35) (Fig. 11); hind femur, tibia and basitarsus 4.25, 14 and 12 times their maximum width, respectively (85 : 20; 140 : 14; 72 : 6) (Figs 13, 14); hind basitarsus 0.5 times as long as hind tibia (72 : 140); and 1.1 times as long as hind tarsal segments 2–5 combined (72 : 68); second segment of hind tarsus 0.4 times as long as basitarsus (38 : 72); fourth segment 0.5 times as long as fifth tarsal segment (without pretarsus) (7 : 14).

*Metasoma*. Metasoma 1.4 times as long as head and mesosoma combined (69 : 48); first metasomal tergite with large dorsope (Fig. 10); first tergite 2.3 times as long as its maximum width (86 : 38) (Fig. 10), 2.5 times as long as propodeum (86 : 35); second tergite with U-shaped medial area, emarginated by rather wide and crenulate lateral furrows fused with suture between second and third tergites (Fig. 12); medial length of second tergite 0.98 times its basal width (47 : 48), 1.1 times medial length of third tergite (47 : 43) (Fig. 12); ovipositor sheath slightly longer than body, and 1.4 times longer than fore wing (120 : 86).

*Sculpture and pubescence*. Frons rugose; vertex finely transversely striate; temple finely rugose-punctate; face largely rugose; clypeus and malar space with dense long setae; malar space largely rugose-punctate contrasting to rather smooth area between hypostomal carina and malar space; notauli largely rugose (Fig. 5); median and lateral lobes of mesoscutum rugose-coriaceous; scutellum finely and densely punctate; pronotum granulate ventrally, rugose dorsally; mesopleuron coriaceous; subalar depression with oblique rugosities; metapleuron setose, largely rugose; propodeum foveolate-rugose (Fig. 7); hind coxa finely and densely punctate laterally, finely rugose-punctate dorsally; first metasomal tergite and medial area of second tergite foveolate-rugose; third tergite largely rugose in basal 0.7, smooth in apical 0.3; fourth tergite largely rugose basally and laterally, finely rugose medially and almost smooth with sparse punctures apically; fifth–sixth tergites finely rugose-punctate basally and laterally, almost smooth apically; ovipositor sheath covered with short dense setae.

*Colour*. Black body; antenna brown; head mainly dark brown, subalar space brownish yellow; palpi white; fore and middle legs yellow, except coxa, trochanters and trochantellus cream white; hind coxa and femur black, trochanters and trochantellus yellow, hind tibia (except yellow basal area) and tarsus infuscate; tegula yellow; fore wing subhyaline, its veins brown, outside area of vein r beneath pterostigma brownish; ovipositor sheath brown.

#### Male.

Unknown.

#### Etymology.

The new species is named after the country (Vietnam) where the holotype was collected.

#### Distribution.

North-eastern Vietnam (Bac Giang Province).

### Key to *Rasnitsynoryctes* species

**Table:** 

1	Vertex without medial longitudinal depression. Propodeum areolate-rugose, with delineated wide areola (Belokobylskij, 2011: fig. 8); first metasomal tergite 1.9 times as long as its apical width (Belokobylskij, 2011: fig. 28); median length of second tergite 0.7 times its basal width; hind wing with five hamuli (Belokobylskij, 2011: fig. 18); hind coxa light brown (Belokobylskij, 2011: fig. 20); hind trochanter pale brown; hind tibia mainly dark (Belokobylskij, 2011: fig. 19). Malaysia	***Rasnitsynoryctesalexandri* Belokobylskij**
–	Vertex with medial longitudinal depression. Propodeum foveolate-rugose, without delineated areola (Fig. 7); first metasomal tergite 2.3 times as long as its apical width (Fig. 10); median length of second tergite almost equal to its basal width (Fig. 12); hind wing with six hamuli (Fig. 16); hind coxa dark brown to black (Figs 11); hind trochanter whitish (Fig. 13); hind tibia mainly pale yellow, infuscate apically (Fig. 14). Vietnam	***Rasnitsynoryctesvietnamicus* sp. nov.**

## Conclusions

The discovery of a new species from very rare Oriental genus *Rasnitsynoryctes* supports the opinion that our knowledge of the tropical and subtropical faunas of the parasitoid wasps is very incomplete even for such large-sized specimens (more than 10.0 mm length). Perhaps one of the main reasons for the rarity of such large specimens in a collection is related with peculiarities of their mode of life (preferring the tree canopies), behaviour, and food preferences related with potential hosts habitats. Further investigation of the relict tropical forests and collecting of such parasitoids by different methods and traps (including rearing from the potential hosts in infested plants, especially tree trunks or branches) in numerous habitats may allow to reveal more numbers of such specimens and taxa and to obtain more information about so called “rare” genera and taxa in the tropics.

## Supplementary Material

XML Treatment for Rasnitsynoryctes

XML Treatment for Rasnitsynoryctes vietnamicus
